# The Impact of Smoking on the Association between Perfluoroalkyl Acids (PFAS) and Thyroid Hormones: A National Health and Nutrition Examination Survey Analysis

**DOI:** 10.3390/toxics8040116

**Published:** 2020-12-09

**Authors:** Maaike van Gerwen, Naomi Alpert, Mathilda Alsen, Kimia Ziadkhanpour, Emanuela Taioli, Eric Genden

**Affiliations:** 1 Department of Otolaryngology-Head and Neck Surgery, Icahn School of Medicine at Mount Sinai, New York, NY 10029, USA; mathilda.alsen@mountsinai.org (M.A.); Eric.Genden@mountsinai.org (E.G.); 2 Institute for Translational Epidemiology, Icahn School of Medicine at Mount Sinai, New York, NY 10029, USA; Naomi.alpert@mountsinai.org (N.A.); emanuela.taioli@mountsinai.org (E.T.); 3 Department of Population Health Science and Policy, Icahn School of Medicine at Mount Sinai, New York, NY 10029, USA; 4 Department of Medical Education, Icahn School of Medicine at Mount Sinai, New York, NY 10029, USA; kimia.ziadkhanpour@icahn.mssm.edu; 5 Tisch Cancer Institute, Icahn School of Medicine at Mount Sinai, New York, NY 10029, USA

**Keywords:** thyroid, PFAS, effect modification, smoking

## Abstract

Perfluoroalkyl acids (PFAS) are known endocrine disrupting chemicals, potentially affecting thyroid function. Smoking has been associated with PFAS levels as well as with thyroid function. The impact of smoking on the association between PFAS and thyroid function remains to be elucidated, so the objective was to assess the effect of PFAS exposure on thyroid function in the general population, stratified by smoking status, using the National Health and Nutrition Examination Survey (NHANES). NHANES adult participants who were part of the 2011–2012 laboratory subsample and had PFAS and thyroid function measured were included (*n* = 1325). Adjusted linear regression models and stratified analyses were performed. There was a significant positive association between perfluorooctanesulfonic acid (PFOS) (*p* = 0.003), perfluorononanoic acid (PFNA) (*p* = 0.014), total PFAS (*p* = 0.004) concentrations and free T4 (FT4). No significant associations were found between perfluorooctanoic acid (PFOA), PFOS, perfluorohexane sulfonate (PFHxS), PFNA, total PFAS and total T4 (TT4) or thyroid stimulating hormone (TSH). In non-smokers, a significant positive association was found between PFOS (*p* = 0.003), PFHxS (*p* = 0.034), PFNA (*p* = 0.012), total PFAS (*p* = 0.003) and FT4 while no significant associations were found in smokers. The present study showed that increased PFAS exposure was associated with increased FT4 in non-smokers, while no association was found in smokers. These results confirm that smoking modifies the association between PFAS exposure and thyroid function.

## 1. Introduction

Perfluoroalkyl acids (PFAS) are known endocrine disrupting chemicals with widespread persistence in the environment due to their stable chemical structure [1,2,3]. In addition to their presence in soil and water, PFAS have also been detected in the air and food, leading to an almost complete exposure of the general population (>95%) [4]. In humans, the biological half-life for PFAS following absorption after inhalation and oral exposure is up to 8 years [5,6].

Due to their unique surfactant properties, PFAS were extensively used in surface coating and protectant formulations, including in paper and cardboard packaging products, carpets, leather products, firefighting foams, paints, and textiles in order to enhance water, grease, and soil repellency [7]. Since the early 2000s, when major American chemical companies voluntarily agreed to eliminate the use of perfluorooctanoic acid (PFOA) and PFOA-related chemicals, certain PFAS have been phased out and are no longer manufactured in the United States (US). Furthermore, the US Environmental Protection Agency (EPA) has established health advisory levels at 70 parts per trillion for PFOA and perfluorooctanesulfonic acid (PFOS) exposure from drinking water [8]. The European Union (EU) currently has restrictions on PFOS and PFOA under the EU Persistent Organic Pollutants (POPs) regulation (EU, 2019) and the Registration, Evaluation, Authorisation and Restriction of Chemicals (REACH) regulation (EU, 2006). The International Agency for Research on Cancer (IARC) listed PFOA as possibly carcinogenic (category 2B) to humans in 2017 [9]. Although restrictions are in place in the US and Europe, the use of PFOS has increased in China, which is problematic as PFAS are persistent in the environment and possibly transported over a long range [6].

PFAS have been identified as endocrine disrupting chemicals, potentially affecting the thyroid axis and thyroid function [10,11,12,13,14,15,16]. Higher concentrations of PFOA and PFOS were associated with thyroid disease in the general US population and in a large high exposure cohort [17,18]. Wen et al. found increased risk of subclinical hypothyroidism associated with PFAS exposure in men (PFOS) and women (PFOA, PFOS, perfluorohexane sulfonate (PFHxS)), but decreased risk of subclinical hypothyroidism after PFOA exposure in men [11]. Additionally, an association between PFOA exposure and hypothyroidism and a positive trend between estimated cumulative PFOA exposure and incident thyroid cancer was reported in a highly exposed community located near a chemical plant in the Mid-Ohio valley [18,19,20].

Prior studies have reported that there is an association between PFAS exposure and smoking status, with significantly higher levels of PFAS seen in smokers compared to non-smokers [21], as well as that smoking affects the thyroid gland. Active smoking was found to be associated with an inhibitory effect on the thyroid gland resulting in lower thyroid hormone levels in smokers compared to non-smokers [22]. Active smoking was also positively associated with mild thyroid stimulating hormone (TSH) decrease [23].

Although the association between PFAS exposure and thyroid function has been studied, the impact of smoking on this association remains an important topic to elucidate as no studies have been performed to date. The goal of the current study was therefore to assess the effect of PFAS exposure on thyroid function in the general population, stratified by smoking status, using the National Health and Nutrition Examination Survey (NHANES).

## 2. Materials and Methods

### 2.1. Data Source and Study Population

NHANES is a program designed to assess the health and nutritional status of the civilian, non-institutionalized population of the US and is administered by the National Center for Health Statistics. Since 1999, the program has been conducted continuously, with data released in a 2 year cycle. NHANES uses an ongoing, cross-sectional survey design, which combines questionnaires about demographics and health-related behaviors with physical examinations and laboratory tests. The NHANES program was approved by the National Center for Health Statistics Ethics Review Board (protocol #2011-17, effective through 26 October 2017).

For this study, we queried NHANES participants, who were part of the 2011–2012 laboratory subsample. Only those with PFAS measurements and sample weights assigned (*n* = 2144) were included. We limited our study to participants 20 years and older, because information on the presence of thyroid disease and thyroid cancer is not available for younger participants (*n_exc_* = 394). Participants were excluded if they were using thyroid medication (*n_exc_* = 113), had thyroid disease or thyroid cancer (*n_exc_* = 63), or were pregnant (*n_exc_* = 28). The study population was further limited to participants who had thyroid function and PFAS measurements recorded (*n_exc_* = 180). Lastly, we excluded participants with outlier thyroid function values of more than three standard deviations away from the mean (*n_exc_* = 41), resulting in a final study population of 1325 participants. Covariates of interest included age, sex, race/ethnicity, body mass index (BMI), iodine status and self-reported smoking status.

### 2.2. NHANES Laboratory Assessments

Detection and quantification of serum PFAS concentrations was achieved using solid phase extraction coupled to high performance liquid chromatography/turbo ion spray ionization-tandem mass spectrometry (online SPE-HPLC-TIS-MS/MS), as described by Kuklenyik et al. [24]. A detailed description of the laboratory methods used can be found in the NHANES 2011–2012 Lab Methods for Polyfluoroalkyl Chemicals. Based on prior literature, we restricted our analysis to four PFAS commonly found in human sera, namely PFOA, PFOS, PFHxS, and perfluorononanoic acid (PFNA) [10,25]. The lower limits of detection (LLOD) for PFOA, PFOS, PFHxS and PFNA were 0.10, 0.20, 0.10, and 0.08 ng/mL, respectively. Values below that were imputed as LLOD/$\sqrt{2}$, consistent with NHANES practice.

Although the NHANES thyroid profile consists of several thyroid function measurements, we restricted our analysis to total thyroxine (TT4), free thyroxine (FT4), total triiodothyronine (T3), free triiodothyronine (FT3) and thyroid stimulating hormone (TSH), as these measurements are referred to in guidelines to determine thyroid function [26]. Thyroid blood specimens were processed and stored at the Collaborative Laboratory Services, Ottumwa (Iowa); a detailed description of the laboratory methods used can be found in the NHANES 2011–2012 Laboratory Procedures Manual.

### 2.3. Statistical Analysis

In order to be representative of the population, NHANES uses a complex, multi-stage, probability sampling strategy. To account for this and produce nationally representative estimates, we used survey procedures and incorporated the survey design variables and weights. All analyses were performed using SAS software, version 9.4 (SAS Institute, Cary, NC, USA) and all statistics shown represent weighted values.

PFAS and thyroid function measurements were natural log (ln-) transformed. The association between PFAS levels and thyroid function measurements was examined using multivariable linear regression models with PFAS as a continuous predictor, adjusted for sex, race/ethnicity, age, BMI, iodine status and smoking status. Age was categorized into age groups (20–39 years, 40–59 years, and 60 years and older). BMI was categorized using the following cut-offs <25 kg/m^2^, 25–30 kg/m^2^ and >30 kg/m^2^, as proposed by the Center for Disease Control and Prevention (CDC). Following the World Health Organization’s recommendations, iodine status was defined as normal (≥100 µg/L urine) and low (<100 µg/L urine). Self-reported smoking status was categorized into smoker (current smoker) and non-smoker (never and former smoker). A sensitivity analysis was performed using serum cotinine levels, instead of self-reporting, to define smoking status. Cotinine, a metabolite of nicotine, is used as a biomarker to distinguish smokers from non-smokers in epidemiological studies [27]. Serum cotinine levels were used to classify smokers and non-smokers using cut off points based on race/ethnicity as described by Benowitz et al. [27]. We assessed the association between thyroid function measurements and the different PFAS (PFOA, PFOS, PFHxS, PFNA) as well as the total PFAS. The total PFAS level was calculated by summing the concentrations of the four separate compounds after converting to µmol/L using molar weights, as described by Buttke et al. [28].

We then stratified the analysis by smoking status to analyze the effect of smoking on the association between PFAS and thyroid function measurements using self-reported smoking status. A sensitivity analysis was performed to test the robustness of the analysis by replacing the self-reported smoking status with serum cotinine levels.

## 3. Results

Of the study population (*n* = 1325), 52.1% were male and 65.5% non-Hispanic white. Most participants were between 40 and 59 years old (40.1%), followed by between 20 and 39 years old (38.2%). Furthermore, 33.1% of the participants had a BMI < 25 kg/m^2^, 34.6% had a BMI of 25–30 kg/m^2^ and 32.3% had a BMI of >30 kg/m^2^. Based on self-report, 20.6% of the sample were smokers, and 25.1% were smokers based on cotinine levels (Table 1).

Across the study population, geometric means (GMs; µmol/L) were 8.9, 33.5, 5.2, and 4.3 for PFOA, PFOS, PFHxS, and PFNA, respectively. The GM for total PFAS for the study population was 55.2 µmol/L, with a concentration of 67.5 µmol/L in males and 44.3 µmol/L in females. PFAS concentrations increased with age; 48.3 53.5, and 73.9 µmol/L for participants of 20–39 years, 40–59 years and 60 years and older, respectively. PFAS concentrations were 58.3 µmol/L and 44.6 µmol/L in non-smoker and smoker, respectively (Table 1). The mean TT4 and FT4 concentrations for the study population were 7.78 µg/dL and 0.82 ng/dL, respectively. The mean TT3 and FT3 concentrations were 114.03 ng/dL and 3.17 ng/mL, respectively; the GM for TSH was 1.50 milli-international units per liter (mIU/L). TSH levels increased with age from 1.42 and 1.47 mIU/L in 20–39 years and 40–59 years old, to 1.72 mIU/L in 60 years and older (Table 1).

There was a significant positive association between PFOS (*p* = 0.003), PFNA (*p* = 0.014), total PFAS (*p* = 0.004) concentrations and FT4 and for PFNA (*p* = 0.024) concentration and FT3. No significant associations were found between PFOA, PFOS, PFHxS, PFNA, total PFAS and TT4 or TSH (Table 2). No interaction was found between PFAS and smoking (*p* = 0.211). Sensitivity analysis adjusting for smoking using serum cotinine showed the same results with a significant positive association for PFOS (*p* = 0.003), PFNA (*p* = 0.010), total PFAS (*p* = 0.004) concentrations and FT4 and for PFNA (*p* = 0.027) concentration and FT3.

A significant positive association was found between PFOS (*p* = 0.003), PFHxS (*p* = 0.034), PFNA (*p* = 0.012), total PFAS (*p* = 0.003) and FT4 in non-smokers while no significant associations were found in smokers (Table 3). A significant positive association was found between PFOA (*p* = 0.021) and PFNA (*p* = 0.015) and TT3 in smokers while no significant association was found in non-smokers (Table 3). Sensitivity analysis using serum cotinine levels showed similar results with a significant positive association between PFOS (*p* = 0.002), PFHxS (*p* = 0.040), PFNA (*p* = 0.026), total PFAS (*p* = 0.003) and FT4 in non-smokers while no significant associations were found in smokers; however, no significant association was found between PFOA (*p* = 0.083), PFNA (0.172) and TT3 in smokers.

## 4. Discussion

The present study is the first study to investigate the effect of smoking on the association between PFAS exposure and thyroid function showing that increased PFAS exposure was associated with increased FT4 in non-smokers, while no association was found in smokers. These results confirm that smoking modifies the association between PFAS exposure and thyroid function, underlining that this is an important issue to be addressed in future studies.

Previous studies have shown that PFAS exposure disrupts the thyroid hormone balance although different results have been reported in different studies. A population-based cohort study by Lin et al., including 551 participants aged between 12 and 30 years, showed a positive association between PFNA levels and FT4 [15], as was found in present study. An analysis including 1181 participants of 20 years and older from NHANES 2007–2010 showed a positive association between PFOA and TT3 in women, a positive association between PFHxS and TT3 and TT4 in women and a negative association between PFHxS and FT4 in men [11]. An NHANES analysis including 1540 participants from 2007–2008 showed no significant association between PFAS and FT3 and FT4; PFOA was positively associated with TT3 and PFHxS was positively associated with TT4 [16]. Webster et al. included 1525 adult participants from the 2007–2008 NHANES cycle and only found a positive association between PFOA and FT3 in participants with normal iodine and thyroid peroxidase antibody levels. No significant associations between PFAS and thyroid hormones were found in participants with low iodine levels [10]. A cross-sectional analysis of 52,296 adults exposed to a year or more of PFAS (PFOA and PFOS) in drinking water and enrolled in the C8 Health Project showed significant elevations of TT4 and reductions in T3 uptake; no significant associations were found with TSH [29]. In 506 male employees enrolled in a fluorochemical medical surveillance program, no association was found between PFOA and TSH or TT4, while a negative association with FT4 and a positive association with TT3 were found [30]. Studies focusing on pregnant women have shown no association between PFAS and thyroid hormones [12,13], except for an increase in TSH associated with increased PFOS levels [13]. Moreover, there are some indications that exposure to certain other endocrine disrupting chemicals, including organochlorine pesticides and polychlorinated biphenyls, may be associated with an increased prevalence of autoimmune thyroiditis among exposed individuals [31,32]; however, future research is needed to investigate if this association also exists for PFAS.

PFAS may affect thyroid hormone levels via multiple mechanisms. PFAS may increase T4 metabolism in the liver and thyroid, replace T4 from serum binding proteins, and reduce T4 production in the thyroid gland. In vitro studies have, for instance, demonstrated that PFAS can compete with T4 for binding to transthyretin, a transport protein that binds approximately 15% of thyroid hormones, which is thought to then slowly increase circulating levels of FT4 [33]. In rats, PFOS has also been shown to increase T4 metabolism and excretion by upregulation of key hepatic transport proteins [34] and increasing T4 glucuronidation and excretion from the liver [35]. Furthermore, it has been shown in rats that PFAS can increase the conversion of T4 to T3 in the thyroid gland by upregulation of the de-iodinase enzyme DIO1 [36].

To date, no studies have investigated the effect of smoking on the association between PFAS exposure and thyroid hormones even though it has been shown in prior studies that smoking affects thyroid function and is inversely associated with thyroid cancer. Smoking is associated with a dose-dependent decrease in TSH in population-based studies [37]. This was demonstrated in a study using NHANES III including 15,592 participants, which showed that smoking appears to be positively associated with a mild TSH decrease [23]. These results were furthermore confirmed by a study including 5639 participants of the 2013–2015 Korean National Health and Nutrition Examination Survey, which found lower TSH levels in current smokers; this finding was even more apparent in iodine-deficient participants [38]. The mechanism by which smoking decreases TSH levels remains unknown [37]; lower TSH levels however may explain why PFAS exposure is not positively associated with FT4 in smokers as it is in non-smokers. Moreover, smokers have a higher prevalence of nontoxic goiter and multiple thyroid nodules, specifically in iodine-deficient areas [37], and smoking is inversely associated with thyroid cancer where the reduced risk is more pronounced for papillary compared to follicular thyroid cancer [37,39,40]. It has been hypothesized that lower BMI and lower TSH levels may contribute to this reduced cancer risk [37]; however, there are also some indications that genetic factors might play a role. A study has demonstrated an inverse association between germline *CYP1A1* inheritance and smoking with risk of papillary thyroid cancer [41]. The results of the current study emphasize the complexity of the interaction between a variety of exposures and the thyroid gland and the need for future studies to investigate the effect of multiple diverse exposures, individually and combined, on thyroid function and thyroid cancer, especially because of the worldwide increase in thyroid cancer [42].

To investigate the association between PFAS exposure and thyroid hormones, we were only able to include the 2011–2012 NHANES lab subsample, in which participants had both PFAS and thyroid hormone measurements completed. Unfortunately, no adjacent sampling cycle measured both PFAS and thyroid hormones in the same lab subsample, therefore limiting our sample size. This study population is however a representative sample of the civilian non-institutionalized US population. Although we were able to adjust for important covariates in our analysis, we were unable to adjust for exposure to other endocrine disrupting chemicals (e.g., polychlorinated biphenyls (PCBs), organochlorines, dioxins, bisphenol A (BPA), polybrominated diphenyl ethers or phthalates), which have been associated with the disruption of thyroid function in prior studies [5], because these chemicals were not all measured in the same lab subsample. Another limitation is the cross-sectional nature of the data as both PFAS and thyroid hormone levels were measured at the same moment in time so reverse causation cannot be ruled out.

## 5. Conclusions

The current study is the first to show that the association between thyroid function, in particular FT4 levels, and PFAS exposure is modified by smoking in adults without a history of thyroid disease. These results may have implications for studies of other endocrine disrupting chemicals whose effects on the thyroid gland may potentially be modified by smoking as well. Furthermore, demonstrating effect modification by a second exposure has implications for future studies investigating the effects of multiple exposures on thyroid function, including smoking, especially for compounds acting via similar pathways.

## Figures and Tables

**Table 1:** Serum perfluoroalkyl acids (PFAS) and thyroid hormone concentrations by participant characteristics in study population (*n* = 1325).

Variables	Total Population	PFOA (µmol/L)	PFOS (µmol/L)	PFHxS (µmol/L)	PFNA (µmol/L)	Total PFAS (µmol/L)	Free T4 (ng/dL)	Total T4 (µg/dL)	Free T3 (pg/mL)	Total T3 (ng/dL)	TSH (mIU/L)
	n (%)	GM (SE)	GM (SE)	GM (SE)	GM (SE)	GM (SE)	Mean (SE)	Mean (SE)	Mean (SE)	Mean (SE)	GM (SE)
All	1325 (100)	8.89 (0.31)	33.52 (1.13)	5.20 (0.26)	4.26 (0.19)	55.18 (1.65)	0.82 (0.01)	7.78 (0.08)	3.17 (0.03)	114.03 (1.26)	1.50 (0.03)
Sex											
Male	717 (52.1)	10.13 (0.37)	42.22 (1.91)	6.91 (0.39)	4.54 (0.27)	67.52 (2.77)	0.82 (0.01)	7.60 (0.09)	3.26 (0.03)	115.14 (1.82)	1.53 (0.04)
Female	608 (47.9)	7.71 (0.38)	26.07 (1.01)	3.81 (0.23)	3.99 (0.14)	44.29 (1.45)	0.82 (0.01)	7.97 (0.10)	3.08 (0.02)	112.82 (1.11)	1.47 (0.04)
Age (years)											
20–39	510 (38.2)	8.18 (0.38)	28.70 (1.55)	4.84 (0.30)	3.95 (0.20)	48.27 (2.43)	0.83 (0.01)	7.78 (0.10)	3.31 (0.03)	120.26 (1.30)	1.42 (0.04)
40–59	442 (40.1)	8.76 (0.47)	32.37 (1.12)	4.80 (0.29)	4.19 (0.27)	53.50 (1.81)	0.80 (0.01)	7.68 (0.10)	3.16 (0.03)	112.19 (1.57)	1.47 (0.08)
≥60	373 (21.7)	10.58 (0.61)	46.97 (2.46)	6.81 (0.52)	5.04 (0.30)	73.89 (3.57)	0.84 (0.01)	7.95 (0.13)	2.96 (0.02)	106.48 (1.57)	1.72 (0.09)
Race											
NHW	469 (65.5)	9.68 (0.46)	35.66 (1.32)	5.97 (0.36)	4.20 (0.25)	58.80 (2.11)	0.81 (0.01)	7.67 (0.10)	3.16 (0.03)	112.82 (1.69)	1.56 (0.05)
NHB	344 (11.5)	7.66 (0.28)	34.17 (3.07)	4.49 (0.20)	4.55 (0.26)	54.26 (3.76)	0.82 (0.01)	8.01 (0.19)	3.16 (0.02)	115.49 (2.24)	1.25 (0.04)
Hispanic/other	512 (23)	7.52 (0.45)	27.85 (2.21)	3.78 (0.30)	4.31 (0.32)	46.44 (3.27)	0.84 (0.01)	7.96 (0.08)	3.22 (0.02)	116.76 (1.33)	1.47 (0.04)
BMI (kg/m^2^)											
<25	446 (33.1)	8.85 (0.42)	32.75 (1.46)	5.19 (0.39)	4.16 (0.20)	54.32 (2.28)	0.83 (0.01)	7.56 (0.11)	3.17 (0.04)	110.89 (1.62)	1.44 (0.04)
25–30	428 (34.6)	9.88 (0.60)	36.36 (2.02)	5.86 (0.39)	4.80 (0.26)	60.64 (2.82)	0.81 (0.01)	7.79 (0.10)	3.15 (0.03)	114.87 (1.52)	1.54 (0.05)
≥30	451 (32.3)	7.98 (0.35)	31.46 (2.10)	4.58 (0.34)	3.84 (0.23)	50.67 (3.03)	0.81 (0.01)	7.98 (0.11)	3.20 (0.03)	116.36 (1.84)	1.51 (0.05)
Smoking *											
Non-smoker	1058 (79.4)	9.30 (0.33)	35.61 (1.27)	5.38 (0.28)	4.47 (0.20)	58.33 (1.86)	0.82 (0.01)	7.79 (0.09)	3.16 (0.02)	113.11 (1.30)	1.52 (0.04)
Smoker	266 (20.6)	7.50 (0.51)	26.56 (1.74)	4.58 (0.35)	3.57 (0.25)	44.63 (2.74)	0.82 (0.01)	7.73 (0.15)	3.24 (0.05)	117.57 (1.92)	1.41 (0.07)
Serum cotinine (ng/mL)											
Non-smoker	993 (74.9)	9.28 (0.33)	35.36 (1.10)	5.35 (0.29)	4.47 (0.04)	58.04 (1.64)	0.82 (0.01)	7.78 (0.09)	3.15 (0.02)	112.91 (1.39)	1.54 (0.03)
Smoker	332 (25.1)	7.82 (0.43)	28.57 (1.90)	4.78 (0.29)	3.70 (0.24)	47.43 (2.71)	0.82 (0.01)	7.78 (0.14)	3.24 (0.05)	117.37 (1.76)	1.38 (0.07)

* Questionnaire data (one missing); BMI: body mass index; GM: geometric mean; NHW: non-Hispanic white; NHB: non-Hispanic black; SE: standard error.

**Table 2:** Weighted adjusted * analysis model estimates for the association between PFAS and thyroid hormones (*n* = 1325).

	Ln TSH (mIU/L)		Ln Total T4 (µg/dL)		Ln Free T4 (ng/dL)		Ln Total T3 (ng/dL)		Ln Free T3 (pg/mL)	
PFAS	B_adj_ * (95% CI)	*p*-Value	B_adj_ * (95% CI)	*p*-Value	B_adj_ * (95% CI)	*p*-Value	B_adj_ * (95% CI)	*p*-Value	B_adj_ * (95% CI)	*p*-Value
Ln PFOA (µmol/L)	0.006 (−0.041; 0.053)	0.787	−0.008 (−0.027; 0.012)	0.411	0.007 (−0.007; 0.021)	0.311	0.011 (−0.009; 0.031)	0.252	0.003 (−0.011; 0.018)	0.612
Ln PFOS (µmol/L)	−0.002 (−0.055; 0.051)	0.943	−0.010 (−0.024; 0.004)	0.138	0.021 (0.008; 0.035)	**0.003**	0.001 (−0.015; 0.017)	0.873	0.002 (−0.006; 0.010)	0.565
Ln PFHxS (µmol/L)	0.002 (−0.036; 0.040)	0.899	−0.005 (−0.023; 0.013)	0.558	0.011 (−0.003; 0.025)	0.127	0.010 (−0.006; 0.026)	0.198	−0.001 (−0.015; 0.012)	0.834
Ln PFNA (µmol/L)	−0.005 (−0.050; 0.041)	0.837	0.000 (−0.019; 0.020)	0.968	0.021 (0.005; 0.038)	**0.014**	0.013 (−0.006; 0.032)	0.174	0.012 (0.002; 0.023)	**0.024**
Ln total PFAS (µmol/L)	−0.004 (−0.056; 0.049)	0.883	−0.010 (−0.027; 0.007)	0.216	0.023 (0.009; 0.038)	**0.004**	0.003 (−0.014; 0.020)	0.716	0.003 (−0.008; 0.014)	0.613

* Adjusted for sex, race, age group, BMI group, iodine status and smoking status (questionnaire); CI: confidence interval, Ln: Natural log transformed; Bold means significant.

**Table 3:** Stratified weighted adjusted * analysis model estimates for the association between PFAS and thyroid hormones in smokers and non-smokers (*n* = 1325).

**Smoking Status**	**Ln TSH (mIU/L)**		**Ln Total T4 (µg/dL)**		**Ln Free T4 (ng/dL)**		**Ln Total T3 (ng/dL)**		**Ln Free T3 (pg/mL)**	
**Non-Smoker**	B_adj_ * (95% CI)	***p*-Value**	B_adj_ * (95% CI)	***p*-Value**	B_adj_ * (95% CI)	***p*-Value**	B_adj_ * (95% CI)	***p*-Value**	B_adj_ * (95% CI)	***p*-Value**
Ln PFOA (µmol/L)	−0.001 (−0.050; 0.049)	0.975	−0.007 (−0.025; 0.011)	0.435	0.010 (−0.005; 0.025)	0.179	0.007 (−0.015; 0.029)	0.494	0.003 (−0.013; 0.020)	0.658
Ln PFOS (µmol/L)	−0.023 (−0.067; 0.021)	0.299	−0.010 (−0.024; 0.005)	0.170	0.024 (0.009; 0.039)	**0.003**	−0.006 (−0.021; 0.00)	0.447	−0.001 (−0.009; 0.008)	0.840
Ln PFHxS (µmol/L)	−0.015 (−0.057; 0.028)	0.478	−0.003 (−0.018; 0.012)	0.675	0.014 (0.001; 0.026)	**0.034**	0.010 (−0.005; 0.024)	0.178	−0.000 (−0.013; 0.012)	0.994
Ln PFNA (µmol/L)	0.005 (−0.047; 0.056)	0.855	0.002 (−0.021; 0.025)	0.866	0.026 (0.006; 0.045)	**0.012**	0.007 (−0.015; 0.029)	0.509	0.013 (−0.002; 0.027)	0.094
Ln PFAS (µmol/L)	−0.021 (−0.066; 0.025)	0.351	−0.010 (−0.025; 0.006)	0.205	0.026 (0.010; 0.043)	**0.003**	−0.004 (−0.021; 0.013)	0.645	0.000 (−0.011; 0.012)	0.938
**Smoker**	B_adj_ * (95% CI)	***p*-Value**	B_adj_ * (95% CI)	***p*-Value**	B_adj_ * (95% CI)	***p*-Value**				
Ln PFOA (µmol/L)	0.051 (−0.073; 0.175)	0.396	−0.016 (−0.058; 0.025)	0.424	−0.013 (−0.042; 0.017)	0.385	0.029 (0.005; 0.054)	**0.021**	0.002 (−0.025; 0.028)	0.904
Ln PFOS (µmol/L)	0.087 (−0.041; 0.214)	0.170	−0.016 (−0.057; 0.025)	0.413	0.004 (−0.021; 0.028)	0.746	0.032 (−0.002; 0.065)	0.064	0.013 (−0.007; 0.034)	0.180
Ln PFHxS (µmol/L)	0.074 (−0.033; 0.180)	0.164	−0.019 (−0.062; 0.024)	0.365	−0.005 (−0.035; 0.025)	0.707	0.015 (−0.023; 0.052)	0.413	−0.003 (−0.029; 0.022)	0.786
Ln PFNA (µmol/L)	−0.023 (−0.129; 0.082)	0.649	−0.008 (−0.048; 0.031)	0.661	0.006 (−0.028; 0.039)	0.726	0.027 (0.006; 0.048)	**0.015**	0.009 (−0.007; 0.026)	0.251
Ln PFAS (µmol/L)	0.074 (−0.055; 0.203)	0.244	−0.020 (−0.066; 0.027)	0.389	0.003 (−0.023; 0.029)	0.808	0.033 (−0.000; 0.067)	0.054	0.011 (−0.012; 0.034)	0.333

* Adjusted for sex, race, age group, BMI group, and iodine status; CI: confidence interval, Ln: Natural log transformed; Bold means significant.
